# Expression and Prognostic Characteristics of m^6^ A RNA Methylation Regulators in Breast Cancer

**DOI:** 10.3389/fgene.2020.604597

**Published:** 2020-12-10

**Authors:** Bo Zhang, Yanlin Gu, Guoqin Jiang

**Affiliations:** General Surgery Department, The Second Affiliated Hospital of Soochow University, Suzhou, China

**Keywords:** N6-methyladenosine (m^6^A) RNA methylation, breast carcinoma, post-transcriptional modification, bioinformatics analysis, prognosis

## Abstract

**Purpose:**

N6-methyladenosine (m^6^A) is the most prevalent modification in mRNA methylation which has a wide effect on biological functions. This study aims to figure out the efficacy of m^6^A RNA methylation regulator-based biomarkers with prognostic significance in breast cancer.

**Patients and Methods:**

The 23 RNA methylation regulators were firstly analyzed through ONCOMINE, then relative RNA-seq transcriptome and clinical data of 1,096 breast cancer samples and 112 normal tissue samples were acquired from The Cancer Gene Atlas (TCGA) database. The expressive distinction was also showed by the Gene Expression Omnibus (GEO) database. The gene expression data of m^6^A RNA regulators in human tissues were acquired from the Genotype-Tissue Expression (GTEx) database. The R v3.5.1 and other online tools such as STRING, bc-GeneExminer v4.5, Kaplan-Meier Plotter were applied for bioinformatics analysis.

**Results:**

Results from ONCOMINE, TCGA, and GEO databases showed distinctive expression and clinical correlations of m^6^A RNA methylation regulators in breast cancer patients. The high expression of YTHDF3, ZC3H13, LRPPRC, and METTL16 indicated poor survival rate in patients with breast cancer, while high expression of RBM15B pointed to a better survival rate. Both univariate and multivariate Cox regression analyses revealed that age and risk scores were related to overall survival (OS). Univariate analysis also delineated that stage, tumor (T) status, lymph node (N) status, and metastasis (M) status were associated with OS. From another perspective, Kaplan-Meier Plotter platform showed that the relatively high expression of YTHDF3 and LRPPRC and the relatively low expression of RBM15B, ZC3H13, and METTL16 in breast cancer patients had worse Relapse-Free Survival (RFS). Breast Cancer Gene-Expression Miner v4.5 showed that LRPPRC level was negatively associated with ER and PR expression, while METTL16, RBM15B, ZC3H13 level was positively linked with ER and PR expression. In HER-2 (+) breast cancer patients, the expression of LRPPRC, METTL16, RBM15B, and ZC3H13 were all lower than the HER-2 (−) group.

**Conclusion:**

The significant difference in expression levels and prognostic value of m^6^A RNA methylation regulators were analyzed and validated in this study. This signature revealed the potential therapeutic value of m^6^A RNA methylation regulators in breast cancer.

## Introduction

According to the data GLOBALCAN 2018, breast cancer is the leading cause of cancer-related deaths and the most frequently diagnosed cancer in females with different phenotypes due to genetic and epigenetic diversity (Bray et al., 2018). Benefiting from the early stage diagnosis and treatment, the 5-year relative survival rate of breast cancer patients primarily diagnosed at stage I reaches to nearly 100%, whereas the survival rate of patients first diagnosed at stage IV is only 26% (Miller et al., 2019). Accurate diagnosis, detection and treatment are always effective approaches to improve the prognosis of breast cancer patients. In breast cancer research, epitranscriptome has been focused for its important role in biological functions. The post-transcriptional modifications are widely located in messenger RNA (mRNA) and non-coding RNA (Shi et al., 2019). Until now, over 170 kinds of RNA modifications have been identified (Boccaletto et al., 2018). The RNA methylated modifications occurring on adenosine present in different forms, such as N6-methyladenosine (m^6^A), N6-2′-O-dimethyladenosine (m^6^Am), N1-methyladenosine (m^1^A). In the 1970s, N6-methyladenosine (m^6^A) was first identified as the most prevalent modification in mRNA. It has been proved to be a reversible and widespread internal adenosine modification particularly enriched in 3′ UTRs codon of mammalian mRNA (Meyer et al., 2012). m^6^A also has a cooperative effect on RNA functions, such as stability, metabolism, transcriptional regulation and intracellular signaling (Roundtree et al., 2017). The biological importance of m^6^A can be embodied in embryonic self-renewal capability (Wang et al., 2014), hematopoietic stem/progenitor cells (HSPCs) emergence (Zhang et al., 2017), circadian control (Fustin et al., 2018), heat shock response (Zhou et al., 2015), neuronal functions (Lence et al., 2016), and tumorigenesis (Lan et al., 2019).

RNA modification is mediated by a series of interplays including “writers” (methyltransferases), “readers” (binding proteins), and “erasers” (demethylases) which regulate a complicated biological regulation process. The “writers” include methyltransferase-like 3 (METTL3), methyltransferase-like 14 (METTL14), methyltransferase-like 16 (METTL16), William tumor 1 associated protein (WTAP), vir like m^6^A methyltransferase associated (VIRMA, also named KIAA1429), zinc finger CCCH domain-containing protein 13 (ZC3H13), RNA binding motif protein 15 (RBM15), and RNA binding motif protein 15B (RBM15B). The “readers” are composed of the YTH family: YTH domain containing 1 (YTHDC1), YTH domain containing 2 (YTHDC2), YTH domain-containing family protein 1 (YTHDF1), YTH domain-containing family protein 2 (YTHDF2), YTH domain-containing family protein 3 (YTHDF3), heterogeneous nuclear ribonucleoprotein C (HNRNPC), FMRP translational regulator 1 (FMR1), leucine-rich pentatricopeptide-repeat containing (LRPPRC), heterogeneous nuclear ribonucleoprotein A2/B1 (HNRNPA2B1), insulin like growth factor binding proteins (IGFBPs): IGFBP1, IGFBP2, IGFBP3 and heterogeneous nuclear ribonucleoprotein G (HNRNPG, also named RBMX). The “erasers” are fat mass and obesity-associated protein (FTO) and α-ketoglutarate-dependent dioxygenase alkB homolog 5 (ALKBH5) (Warda et al., 2017; Yang et al., 2018). Each m^6^A RNA methylation regulator serves a critical role in the RNA methylation process. Although in the past few years, researches have been done to decipher the interactions coupled with m^6^A RNA methylation regulators, the nature of RNA modifications and their biological functions in breast cancer remain unclear.

To further clarify the expression of various RNA regulators and their impacts on the prognosis of breast cancer, we obtained the expression of 23 m^6^A RNA regulators from ONCOMINE. We downloaded breast cancer datasets from TCGA, which included 1096 breast cancer samples and 112 normal tissue samples to analyze the distinct expressions of 23 RNA methylation regulators and their clinical characteristics in breast cancer. The expressive results were also confirmed by the GEO database. Through the COX regression analysis and the least absolute shrinkage and selection operator (LASSO) regression, we found five m^6^A methylation regulators which were significant to clinical prognosis. Kaplan-Meier Plotter and bc-GenExMiner v4.5 were used to further explore the clinicopathological and prognostic value of the five m^6^A methylation regulators. The Gene Ontology (GO) analysis of 23 regulators and the Gene Set Enrichment Analysis (GSEA) may help to shed light on their biological research value.

## Materials and Methods

### Datasets and Study Cohort

#### ONCOMINE Database

ONCOMINE database^1^ is a systematic and comprehensive aggregation of microarray datasets. The 23 m^6^A RNA methylation regulators were initially analyzed through ONCOMINE by evaluating their expression in various cancer types compared with the normal. We searched each regulator for the Cancer vs. Normal Analysis and typed with the threshold of *p* < 0.05 and with the gene ranking at the top 10%.

#### TCGA Database

The RNA-seq transcriptome data of 1,096 breast cancer samples and 112 normal tissue samples were downloaded from the TCGA database^2^. Clinical data of breast cancer patients was also downloaded from TCGA. All data were normalized by the expectation-maximization method and converted into Sample IDs by Perl. Patients without survival information were excluded.

#### GEO Database

The differential expression of m^6^A RNA methylation regulators between breast cancer tissues and adjacent normal tissues was verified by GSE70905. The data was downloaded from the GEO database^3^. The GSE70905 included 47 breast adenocarcinoma and 47 paired adjacent normal breast tissues.

#### Kaplan-Meier Plotter

The Kaplan-Meier curves were displayed via Kaplan-Meier Plotter^4^ with the log-rank test. It was performed to provide survival information of m^6^A RNA methylation regulators in breast cancer patients.

#### Breast Cancer Gene-Expression Miner v4.5

A public statistical mining tool, the Breast Cancer Gene-Expression Miner v4.5^5^, was used to evaluate the correlations between mRNA expression and clinicopathological characteristics, such as ER, PR, and HER-2 status, as well as the Nottingham Prognostic Index (NPI) and the Scarff-Bloom-Richardson (SBR) grading.

#### Other Online Databases

The Protein-Protein Interaction (PPI) network was used to exhibit the comprehensive functional annotation of related proteins (version11.0)^6^. Besides, the gene expression of m^6^A RNA methylation regulators in human tissues was acquired from the GTEx database^7^. The data was acquired in a way where strict ethical guidelines were followed.

### Selection of m^6^A Methylation Regulators

A total of 23 regulators were selected according to the previous study, including ALKBH5, FTO, HNRNPC, HNRNPA2B1, KIAA1429, METTL14, METTL3, METTL16, RBM15, RBMX, RBM15B, WTAP, YTHDC1, YTHDC2, YTHDF1, YTHDF2, YTHDF3, ZC3H13, IGF2BP1, IGF2BP2, IGF2BP3, FMR1, and LRPPRC. Then their expressive correlations and clinicopathological characteristics were analyzed.

### Bioinformatic Analysis

The expressive distinction and correlation of 23 regulators were analyzed through “Limma” package by using R v3.5.1^8^ with the cut-off criteria of *p* < 0.05. A heatmap diagram was drawn to compare the expression of m^6^A RNA methylation regulators between tumors and normal tissues. Vioplot diagrams were constructed to visualize the expression of 23 regulators of breast cancer and normal tissues. Besides, GO analysis of 17 regulators was performed through “GO PLOT” and “Digest” package. Univariate Cox regression analysis and LASSO Cox regression model were used to correlate prognostic m^6^A regulators, and the corresponding results showed that five regulators significantly correlated with the prognosis of breast cancer according to the statistical analysis of log-rank *p*-value (*p* < 0.05) and the hazard ratio (HR) with 95% confidence intervals. If the HR > 1, the gene expression shows a positive correlation with OS, while HR < 1 indicates a negative correlation with OS. Additionally, the receiver operating characteristic (ROC) curves were used to evaluate sensitivity and specificity. Next, all the samples selected by LASSO analysis were divided into two groups judged by the risk score (RS). Patients with RS above the mean were assigned to the high-risk group while the rest patients with RS below the mean were assigned to the low-risk groups. The GSEA was performed to study the functions m^6^A RNA methylation regulators by using “kegg.v7.1 symbol.gmt” package.

### Statistical Analysis

The expression of m^6^A RNA methylation regulators between breast cancer and normal tissues were compared through the Wilcoxon test. The high-risk group and the low-risk group were classified according to the median risk score. The chi-square test was used to compare the relationship between the clinicopathological variables and risk score. Cox univariate and multivariate analyses were performed to compare the relationship between clinicopathological variables and risk score. The *t*-test was used to compare the difference between the two groups classified by risk scores. The value *p* < 0.05 was considered to be statistically significant.

## Results

### Expression of m^6^A RNA Methylation Regulators in Breast Cancer

**FIGURE 1:**
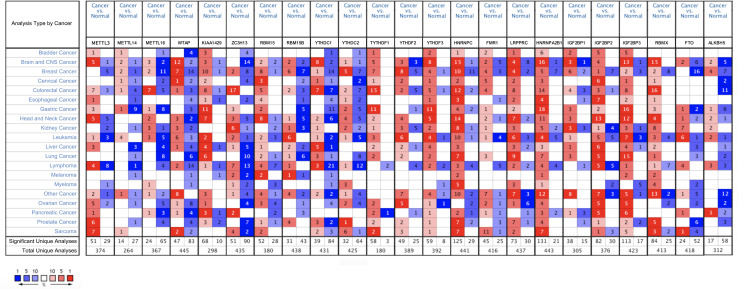
The overall mRNA expression of 23 RNA methylation regulators in various cancer types. The number in the small check signifies the number of datasets which meet the criteria. The red means high expression while the blue means low expression of relative genes in different analyses. And the shade in the box indicates the gene ranks. Statistically significant values are demarcated with the threshold *p* < 0.05.

**FIGURE 2:**
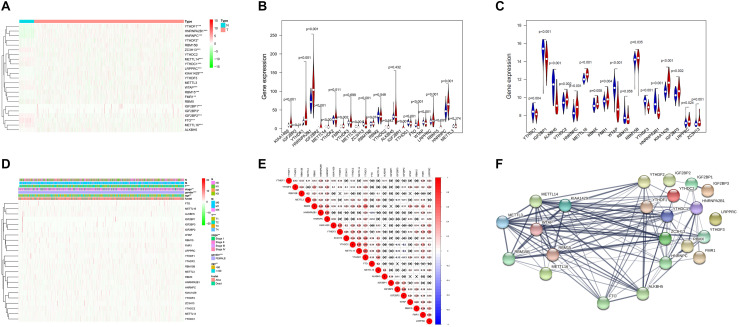
Expression and correlation of m^6^A RNA methylation regulation factors in breast cancer. **(A)** Expression heatmap of 23 m^6^A RNA regulators in breast cancer tissues and normal tissues. Vioplot demonstrates the expression of the regulation factors with the comparison between breast cancer tissues and normal tissues through the data from The Cancer Gene Atlas (TCGA) **(B)** and Gene Expression Omnibus (GEO) **(C)**. The white spot in every “violin” presents the median expression level. **(D)** The correlations between m^6^A RNA methylation regulators and clinicopathological features. **(E)** Spearman correlation analysis of m^6^A RNA methylation regulators. **(F)** The PPI network of m^6^A RNA methylation regulators. *p < 0.05, **p < 0.01, ***p < 0.001.

The ONCOMINE analysis revealed the gene expression of the 23 RNA methylation regulators in different types of cancer compared with normal tissues (Figure 1). From the ONCOMINE database, we had a brief view of the expression of the genes. METTL3, METTL16, ZC3H13, YTHDC1 and FTO were expressed at a low level in breast cancer, while KIAA1429, RBM15, and YTHDF1 were expressed at a high level. Then the m^6^A RNA methylation regulators were analyzed to compare the expression level through the data from the TCGA database. The expression levels in each regulator were compared by the average level of all tissue samples. YTHDF1 (*p* < 0.001), HNRNPA2B1 (*p* < 0.001), HNRNPC (*p* < 0.001), LRPPRC (*p* < 0.001), KIAA1429 (*p* < 0.001), RBM15 (*p* < 0.001), FMR1 (*p* < 0.01), IGF2BP1 (*p* < 0.01), YTHDF2 (*p* < 0.05), and IGF2BP3 (*p* < 0.05) were over-expressed at the mean level in breast cancer tissues compared with normal tissues, while ZC3H13 (*p* < 0.001), METTL14 (*p* < 0.001), YTHDC1 (*p* < 0.001), WTAP (*p* < 0.001), IGF2BP2 (*p* < 0.001), FTO (*p* < 0.001), and METTL16 (*p* < 0.001) were found to be under-expressed in breast cancer tissues in comparison with normal tissues (Figures 2A,B). In GSE70905, YTHDC1 (*p* < 0.05), IGFBP1 (*p* < 0.001), ALKBH5 (*p* < 0.001), WTAP (*p* < 0.001), YTHDF3 (*p* < 0.001) and HNRNPA2B1 (*p* < 0.001) were down-regulated in breast cancer to the adjacent. While YTHDC2 (*p* < 0.05), HNRNPC (*p* < 0.001), METTL16 (*p* < 0.001), RBMX (*p* < 0.05), FMR1 (*p* < 0.05), RBM15B (*p* < 0.05), KIAA1429 (*p* < 0.001), IGFBP3 (*p* < 0.05), LRPPRC (*p* < 0.05) and ZC3H13 (*p* = 0.001) were up-regulated (Figure 2C). The correlations between m^6^A RNA methylation regulators and clinicopathological features, such as N status, M status, T status, pathological stage, age, and survival state were investigated. The T status (*p* < 0.001), stage (*p* < 0.01), and age (*p* < 0.01) were found to be relevant to the expression level of m^6^A RNA methylation regulators (Figure 2D). Spearman correlation analysis was performed to compare the relationship between each other. YTHDC1 and METTL14 showed the most significant positive correlation (Figure 2E). The PPI network showed from a novel perspective that the regulators were strongly linked with each other and the line sickness intuitively disclosed the strength of interactions (Figure 2F).

### Expression and Biological Function Annotation of m^6^A RNA Methylation Regulators

**FIGURE 3:**
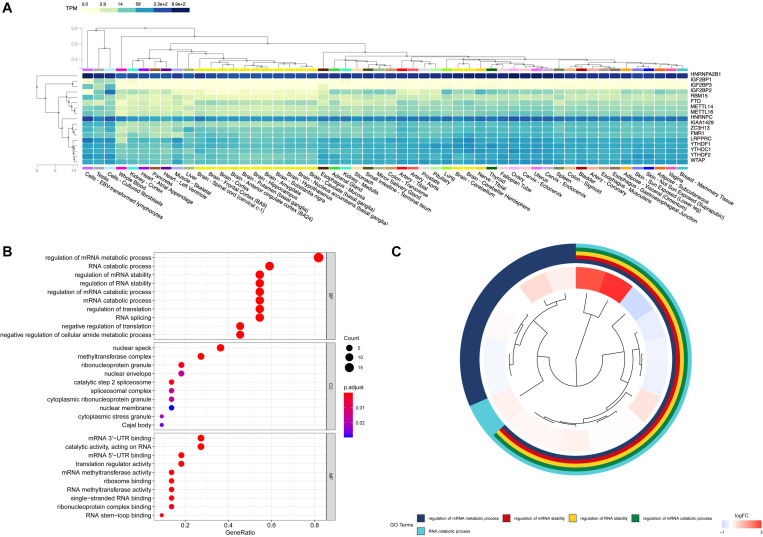
Expression and biological functional annotation of m^6^A RNA methylation regulators. **(A)** The expression levels of 17 m^6^A RNA regulators in normal tissues from the Genotype-Tissue Expression (GTEx) database. **(B,C)** The Gene Ontology (GO) terms of the genes, including molecular function, biological process, and cellular components.

In order to figure out the expression of RNA methylation regulators in normal human tissues, the GTEx database was used as a supplementary data to verify their research value. On a broad range of tissue types, HNRNPA2B1, HNRNPC, LRPPRC, and YTHDF2 had correspondingly high expression in breast tissues. The regulators such as IGFBP1, IGFBP3 were relatively low by contrast (Figure 3A). The GO analysis showed that these m^6^A RNA methylation regulators participated in the regulation of mRNA metabolic process, the RNA catabolic process and the regulation of mRNA stability (Figures 3B,C).

### Prognostic Value and Risk Signature of m^6^A RNA Methylation Regulators

**FIGURE 4:**
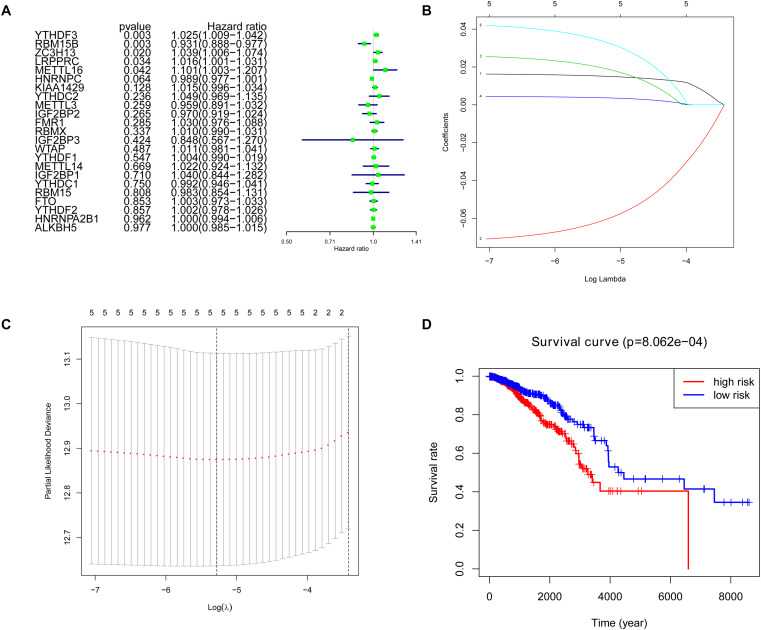
Selection of clinical-pathological m^6^A RNA regulators with prognostic value. **(A)** Univariate Cox regression analysis showed the *p*-value, hazard ratio (HR), and 95% confidence interval (CI) of 23 m^6^A RNA methylation regulators. **(B,C)** Five regulators were selected for risk coefficient calculation through the least absolute shrinkage and selection operator (LASSO) Cox regression. **(D)** Kaplan-Meier Curve showed the overall survival of high and low-risk groups according to the risk score.

Cox univariate analysis was performed to select the m^6^A RNA methylation regulators that were associated with prognosis. The results showed that 5 out of 23 regulators had a close correlation with overall survival (OS). High expression of YTHDF3 (HR: 1.025, 95% CI: 1.009–1.042), ZC3H13 (HR: 1.039, 95% CI: 1.006–1.074), LRPPRC (HR: 1.016, 95% CI: 1.001–1.031) and METTL16 (HR: 1.101, 95% CI: 1.003–1.207) indicated poor survival in patients with breast cancer, while high expression of RBM15B (HR: 0.931, 95% CI: 0.888–0.977) corresponds with better survival (Figure 4A). To figure out the most significant prognostic regulators, RBM15B, YTHDF3, ZC3H13, LRPPRC, and METTL16 were selected with the criteria of *p* < 0.05 to perform LASSO Cox regression algorithm. Results showed that these five regulators were all strong prognostic factors. Then RBM15B, YTHDF3, ZC3H13, LRPPRC, and METTL16 were selected to evaluate risk characteristics (Figures 4B,C). We divided patients into the low-risk group and the high-risk group according to the median risk score based on the coefficients from LASSO analysis. The survival analysis of the five-gene risk signature demonstrated that the high-risk group patients had a significantly poorer prognosis than the low-risk patients (*p* < 0.001) (Figure 4D).

### Relationship Between Five m^6^A RNA Methylation Regulators and Clinicopathological Features of Breast Cancer

**FIGURE 5:**
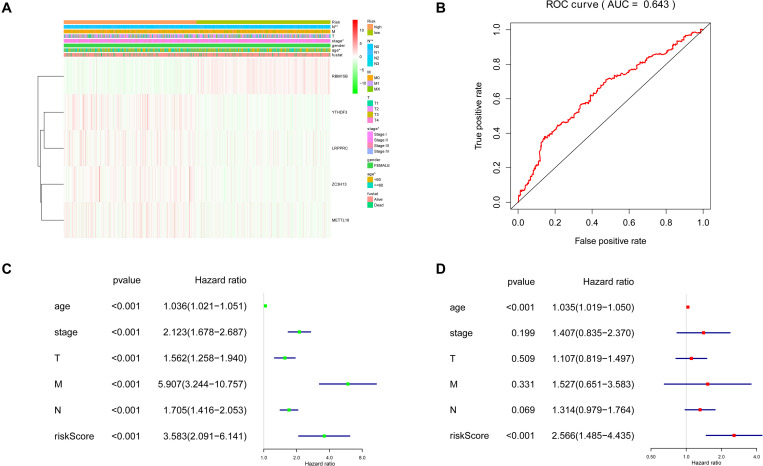
Clinicopathological features of the two risk subgroups based on five selected regulators. **(A)** The heatmap shows the expression levels of five m^6^A RNA regulators in high and low-risk groups. **(B)** The receiver operating characteristic (ROC) curves were used to validate the predictive specificity and sensitivity. The association between clinicopathological factors and overall survival of breast cancer patients through univariate **(C)** and multivariate **(D)** Cox regression analyses.

**FIGURE 6:**
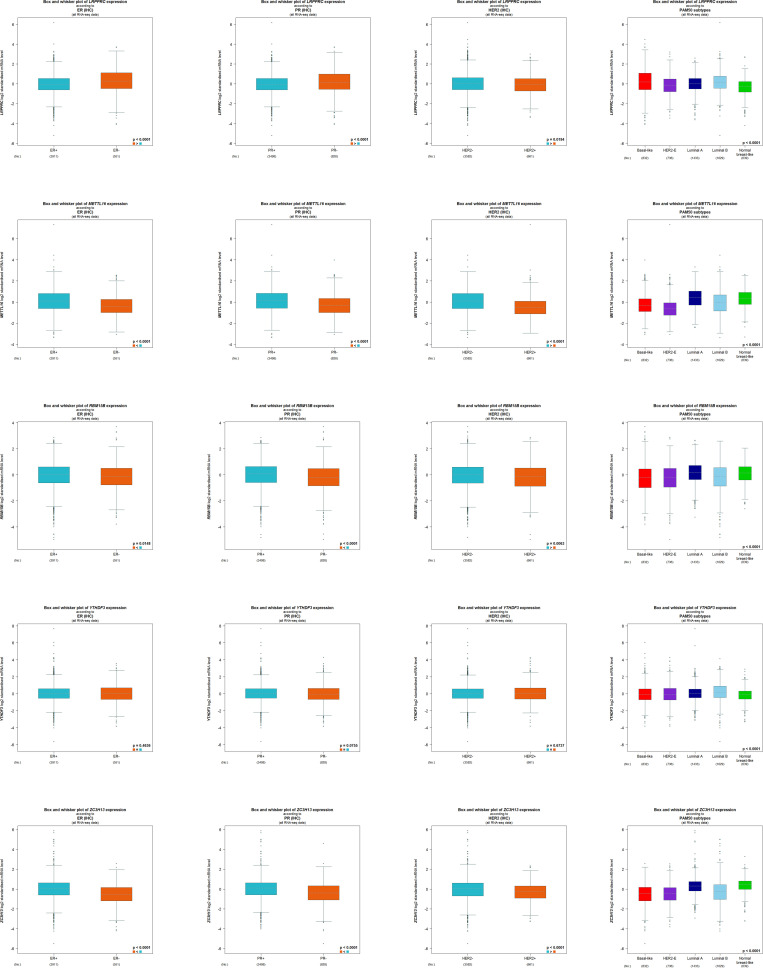
RNA-seq data from bc-GenExMiner v4.5 showing the comparison outcomes with ER, PR, HER-2, and PAM50 subtypes on the expression level of YTHDF3, RBM15B, ZC3H13, LRPPRC, and METTL16.

**FIGURE 7:**
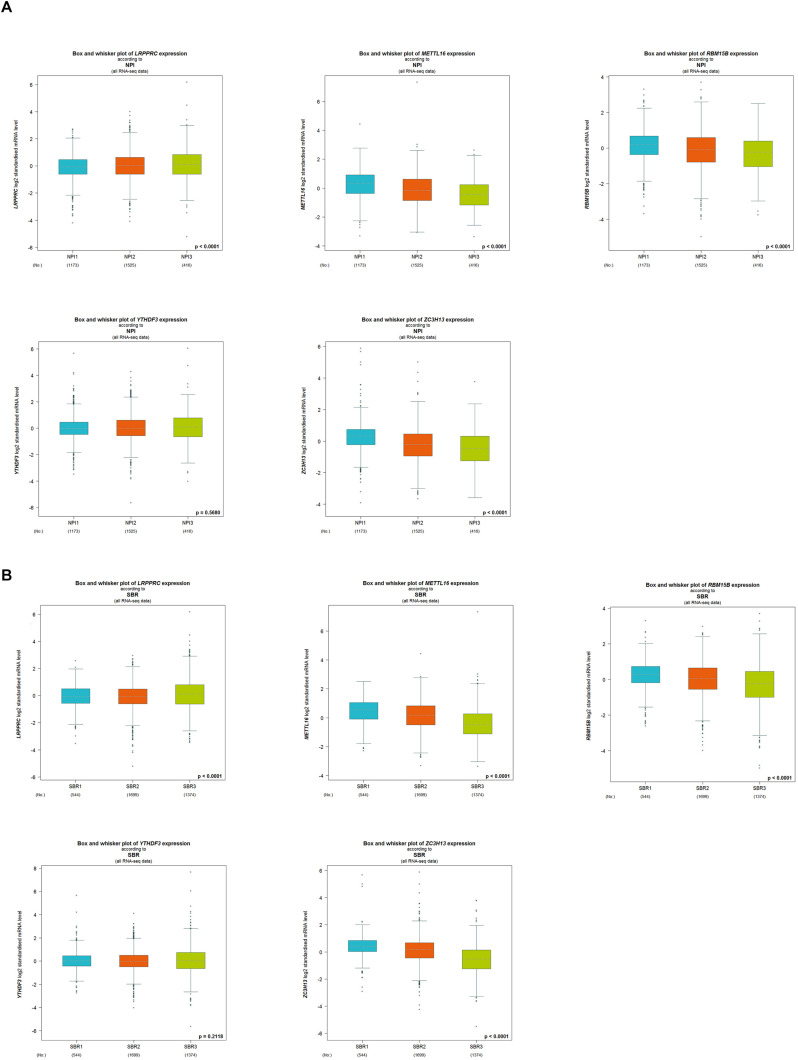
The correlation between the expression level of LRPPRC, METTL16, RBM15B, YTHDF3, and ZC3H13 and the Nottingham prognostic index (NPI) **(A)** and the Scarff-Bloom-Richardson (SBR) **(B)** grading. Patients with higher NPI and SBR grade tend to have higher expression of LRPPRC and lower expression of METTL16, RBM15B, and ZC3H13 (*p* < 0.0001).

**FIGURE 8:**
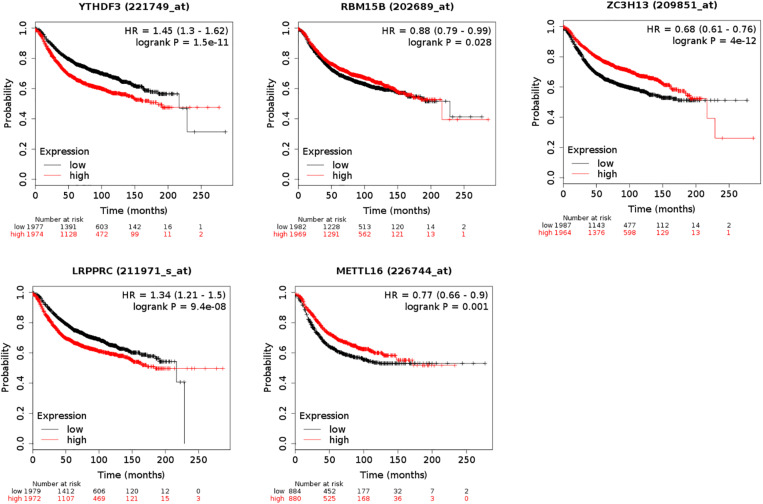
Kaplan-Meier Plotter showing the prognostic value of five m^6^A RNA methylation regulators on Relapse-Free Survival (RFS) in breast cancer patients. Breast cancer patients with relatively high expression of YTHDF3 and LRPPRC as well as relatively low expression of ZC3H13, METTL16, and RBM15B presented with worse RFS.

The five regulators of m^6^A RNA methylation regulators vary differently among clinicopathological characteristics (Figure 5A). When the data was divided into high-risk and low-risk groups, it can be observed that RBM15B was in low expression, while YTHDF3, LRPPRC, ZC3H13, and METTL16 were in high expression in the high-risk group. We further performed the receiver operating characteristic (ROC) curve to evaluate the predictive specificity and sensitivity, and the area under the curve (AUC) was determined to be 0.643 (Figure 5B). Both univariate and multivariate Cox regression analyses indicated that age and risk scores were related to OS. Univariate analyses also revealed that stage, T status, N status, and M status were associated with OS (Figures 5C,D). After retrieving the RNA-seq data including the TCGA and SCAN-B databases, we got the comparison outcomes with ER, PR, HER-2 and PAM50 subtypes (Figure 6). LRPPRC was negatively associated with ER and PR expression, while METTL16, RBM15B, and ZC3H13 were positively associated with ER and PR expression. In HER-2 (+) breast cancer patients, LRPPRC, METTL16, RBM15B, and ZC3H13 expressed lower than the HER-2 (−). LRPPRC showed the highest expression in basal-like breast cancer subtype. METTL16 expressed at a lower level in HER-E and basal-like subtypes. RBM15B expressed higher in Luminal A and normal breast-like subtypes of breast cancer. ZC3H13 expressed lower in basal-like, HER-E and Luminal B subtypes than in Luminal A and basal-like. However, no obvious difference among these five subtypes was observed with regards to YTHDF3 expression. These results were also verified by METABRIC database in bc-GenExMiner v4.5 (Supplementary Figure 1). LRPPRC and YTHDF3 were negatively associated with ER and PR expression, while METTL16 and RBM15B were positively associated with ER and PR expression. In HER-2 (+) breast cancer patients, METTL16 expressed lower than the HER-2 (−), but YTHDF3 expressed higher than the HER-2 (−). And we got similar results of PAM50 subtypes from METABRIC database. As a prognostic factor in breast cancer, the NPI and SBR histological grade is widely applied to predict tumor prognosis. Patients with high NPI and SBR grade tend to have a poor prognosis. Results from bc-GenExMiner v4.5 showed that patients with higher NPI and SBR grade tended to have higher expression of LRPPRC and lower expression of METTL16, RBM15B, and ZC3H13 (Figure 7). These results were also confirmed by METABRIC database in bc-GenExMiner v4.5 (Supplementary Figure 2). Patients with higher NPI and SBR grade tend to have higher expression of LRPPRC and YTHDF3 and lower expression of METTL16 and RBM15B. The prognostic value of m^6^A RNA methylation regulators on RFS in breast cancer patients was analyzed via the Kaplan-Meier Plotter platform. The results in breast cancer patients showed that the relatively high expression of YTHDF3 and LRPPRC were remarkably associated with worse RFS, whereas relatively high expression of RBM15B, ZC3H13, and METTL16 had better RFS (Figure 8).

### Associated Biological Pathways of Five m^6^A RNA Methylation Regulators

**FIGURE 9:**
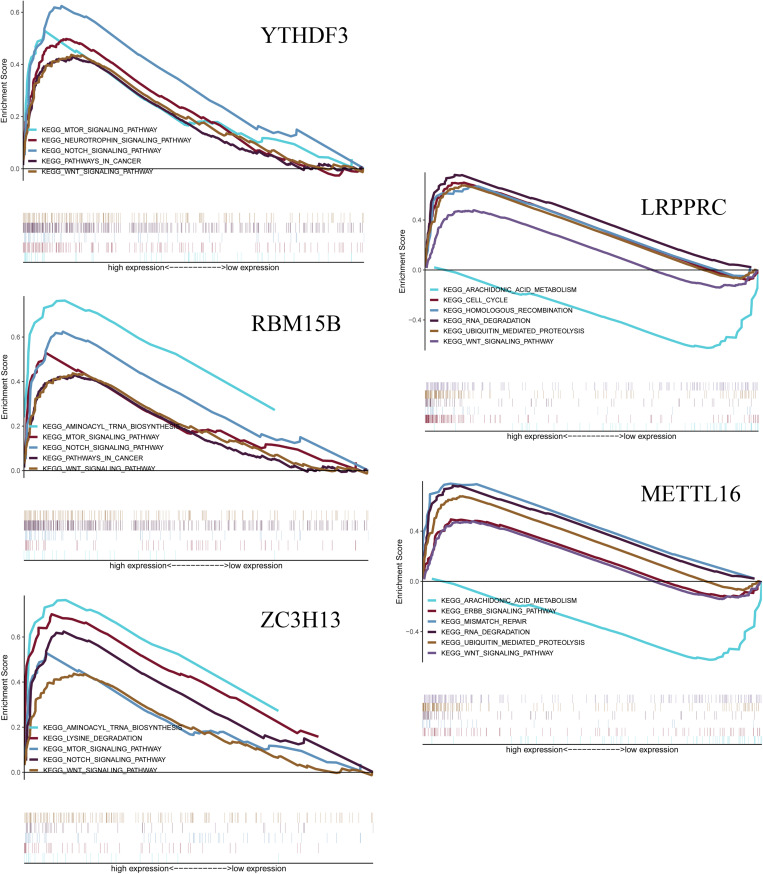
Gene Set Enrichment Analysis (GSEA) shows that the five m^6^A RNA methylation regulators, including YTHDF3, RBM15B, ZC3H13, LRPPRC, METTL16. The results are based on their normalized enrichment score (NES) and normalized *p*-value. Pathways with positive NES are positively correlated with the genes, and pathways with negative NES are negatively correlated with the genes.

The GSEA analysis was performed to identify associated pathways. We selected significantly enriched signaling pathways based on their normalized enrichment score (NES) and normalized *p*-value (Figure 9). YTHDF3 was enriched in mTOR signaling pathway (NES = 1.98, *p* = 0.021), neurotrophin signaling pathway (NES = 1.98, *p* = 0.023), Notch signaling pathway (NES = 2.10, *p* = 0.012), pathways in cancer (NES = 1.84, *p* = 0.047) and Wnt signaling pathway (NES = 1.86, *p* = 0.042). RBM15B was enriched in aminoacyl tRNA biosynthesis (NES = 2.12, *p* = 0.011), mTOR signaling pathway (NES = 1.94, *p* = 0.023), Notch signaling pathway (NES = 2.10, *p* = 0.008), pathways in cancer (NES = 1.82, *p* = 0.049), and Wnt signaling pathway (NES = 1.86, *p* = 0.044). ZC3H13 was enriched in aminoacyl tRNA biosynthesis (NES = 2.13, *p* = 0.005), lysine degradation (NES = 2.39, *p* < 0.001), mTOR signaling pathway (NES = 1.95, *p* = 0.024), Notch signaling pathway (NES = 2.15, *p* = 0.005), and Wnt signaling pathway (NES = 1.87, *p* = 0.038). LRPPRC was enriched in cell cycle (NES = 2.28, *p* = 0.002), homologous recombination pathway (NES = 1.95, *p* = 0.017), RNA degradation (NES = 2.64 *p* < 0.001), ubiquitin-mediated proteolysis (NES = 2.63, *p* < 0.001), and Wnt signaling pathway (NES = 2.01, *p* = 0.015). LRPPRC was down-regulated in arachidonic acid metabolism (NES = −2.31, *p* = 0.002). METTL16 was enriched in ErbB signaling pathway (NES = 1.96, *p* = 0.010), mismatch repair (NES = 2.01, *p* = 0.008), RNA degradation (NES = 2.59, *p* < 0.001), and Wnt signaling pathway (NES = 2.39, *p* = 0.004). METTL16 was down-regulated in arachidonic acid metabolism (NES = −2.26, *p* = 0.004).

## Discussion

The 5-year relative survival rate of breast cancer has been improved significantly due to the improvement of earlier diagnosis and treatment. However, there is still a long way to go for new therapeutic targets for breast cancer treatment. The m^6^A RNA methylation is an important post-transcriptional factor that affects tumor occurrence and development. Previous studies show a close relationship between RNA methylation and breast cancer. Hypoxia-inducible factor (HIF)-1α-and HIF-2α-dependent ALKBH5 in breast cancer cells can be stimulated under hypoxia condition, leading to demethylation of NANOG mRNA. Furthermore, the high expression level of NANOG leads to the enrichment of breast cancer stem cells (BCSCs) (Zhang et al., 2016). Cai et al. (2018) reported that the mTORC1 activator mammalian hepatitis B X-interacting protein (HBXIP) can active METTL3 by prohibiting the expression of oncogenes-related miRNA let-7g. While the up-regulated METTL3 can simultaneously promote the HBXIP expression. Thereby, HBXIP, METTL3 and let-7g form a feedback loop accelerating proliferation and invasion of breast cancer. The “writer” KIAA1429 is confirmed to be up-regulated in breast cancer, and 5′-fluorouracil is effective for exhibiting the expression of KIAA1429 and its methylated target cyclin-dependent kinase 1 (CDK1) (Qian et al., 2019). These studies mostly concentrate on few methylation regulators, such as METTL3, METTL14, KIAA1429, FTO, and ALKBH5. With the continuing discovery of novel m^6^A regulators, there is an ever-increasing demand for in-depth research into RNA methylation about breast cancer.

In our present study, we analyzed the expression of the 23 RNA methylation regulators in the round from ONCOMINE database. Based on 1,096 tumor tissues and 112 normal tissues from the TCGA database, the analytic outcomes showed that 17 out of 23 regulators had statistical significance (*p* < 0.05). Surprisingly, some researches have verified that the repression of HNRNPC can inhibit two breast cancer cell lines (MCF7 and T47D) proliferation through the accumulation of double-stranded RNA (dsRNA), the binding ligand of RIG-I, and anti-proliferation activity induced by the stimulated IFN cytokine (Wu et al., 2018). The silencing of the m^6^A methyltransferase has an effect on the stability of gene expression, such as the interference of the TP53 signaling pathway which is a vital tumor suppressor gene (Dominissini et al., 2012). The p53 protein binds closely with HNRNPC and causes destabilization of HNRNPC. Under the treatment of doxorubicin, lncRNA SNHG1 and HNRNPC can reach a balance on the mediation of p53 (Shen et al., 2017). In line with the TCGA outcomes, up-regulated HNRNPA2B1 is concluded to promote breast cancer tumorigenic potential by activating extracellular-signal-regulated kinase 1/2 (ERK1/2), and signal transducer and activator of transcription 3 (STAT3). Knockdown of HNRNPA2B1 can prohibit proliferation, accelerate apoptosis and prolong S phase *in vitro*, while restraining tumorigenicity *in vivo* (Hu et al., 2017).

The m^6^A RNA methylation regulators are of great significance in the occurrence, development and prognosis of various types of cancers. Recently, by using bioinformatic databases, researchers have discovered its importance in tumor therapies. The variations and/or mutations of m^6^A RNA methylation regulators occurring in the acute myeloid leukemia (AML) patients present a close relation with the mutations of TP53 may disclose the poor prognosis in AML (Kwok et al., 2017). Patients with high levels of mRNA methylation resulting from overexpression of reader proteins and methyltransferase complexes showed poor survival benefits in prostate cancer (Ji et al., 2020). In the subgroup construction signature of TCGA and GEO database, the m^6^A regulators showed a great value in malignant progression and prognosis of uveal melanoma (UM) (Tang et al., 2020).

According to the clinical database of breast cancer of TCGA, RBM15B, YTHDF3, ZC3H13, METTL16, and LRPPRC were picked up indicating strong associations with clinical characteristics. We used a variety of databases to verify their impact on the survival and prognosis of breast cancer patients and its potential research significance. The outcome revealed a clear direction for further study *in vivo* and *in vitro*. RBM15B is the paralog of RBM15, and they are confirmed to bind METTL3 relying on the meditation of WTAP (Patil et al., 2016). BRCA1-associated protein-1 (BAP1) is found to be expressed at a low level in breast cancer patients, while RBM15B has a positive correlation with BAP1 in invasive breast cancer (Shahriyari et al., 2019). YTH domain protein family contains several m^6^A-binding proteins playing parts as readers in m^6^A methylation but with different functions. YTHDF1 and YTHDF3 work cooperatively to promote ribosome loading, while YTHDF2 accelerates the decay of mRNA and YTHDC1 acts as a recruiter for mRNA splicing (Xiao et al., 2016; Hsu et al., 2017; Li et al., 2017). YTHDF3 has been verified to correlate with several kinds of carcinoma, such as colorectal cancer (CRC) (Liu et al., 2019), gastric cancer (Zhang et al., 2019), bladder cancer (Jin et al., 2019), breast cancer (Liu et al., 2019), and so on. In the ONCOMINE and TCGA databases, we didn’t notice a considerable difference in the expression of RBM15B and YTHDF3 in breast cancer. Whether it has an impact on the occurrence, development and prognosis of breast cancer needs to be further confirmed by research. ZC3H13 is a novel “writer” mediating m^6^A methylation. The ablation of ZC3H13 can lead to an obvious decrease of m^6^A enrichment, especially at the 3′end of the mRNA. It is also speculated that ZC3H13 is a component of the RBM15/ZC3H13/WTAP/VIRMA/HAIKAI complex, also refers to the m^6^A-METTL-associated complex, which regulates m^6^A methylation either on its own or interacts with METTL3/METTL14 complex (Knuckles et al., 2018). In the triple-negative subtype of inflammatory breast cancer (TN-IBC), ZC3H13 is down-regulated in contrast to TN-non-IBC (Funakoshi et al., 2019). The outcome of this research is consistent with the results of our bioinformatics analysis. METTL16 was first found to have a close correlation with a cancer-promoting long non-coding RNA: metastasis-associated lung adenocarcinoma transcript 1 (MALAT1) (Brown et al., 2016). As a cancer-promoting factor, MALAT1 is closely related to certain biological characteristics such as vascular epithelialization, migration and distant metastasis, postoperative fever, and systemic inflammatory response in breast cancer (Huang et al., 2018; Li et al., 2018; Gomes et al., 2019). METTL16 is also reported in U6 small nuclear (snRNA), pre-mRNAs and other lncRNA (Warda et al., 2017). METTL16 binds the UACAGAGAA sequence on the 3′ UTR of MAT2A mRNA sequence for MAT2A splicing, the procedure of which can encode the SAM synthetase expression and form a methylation pattern regulated according to SAM level which is quite different from the way that METTL3-METTL14-WTAP complex acts (Pendleton et al., 2017). Former research has pointed out that the biochemical synthesis of SAM is a unique metabolic “soft spot” of triple-negative BCSCs whose drug-resistance can be eradicated by combining methionine depletion with MAT2A suppression (Strekalova et al., 2019). Although these regulators failed to present desired and consistent outcomes in the analysis of expression levels and prognostic values, it’s worth further exploring their biological functions and interactions in breast cancer. Besides, the subtypes of breast cancer may interfere in RNA methylation which indicates new research interests in exploring different subtypes. LRPPRC is an RNA-binding protein regulating mitochondrial DNA-encoded mRNA and also transactivating nuclear DNAs. The immunohistochemical detection of several types of cancers, including lung adenocarcinoma, breast cancer, oesophageal squamous cell cancer, gastric adenocarcinoma, etc., shows that LRPPRC is abundantly expressed in cancer tissues in contrast to the adjacent normal tissues. It can promote apoptosis resistance and invasive activity of cancer cells (Tian et al., 2012). Research on BCSCs shows that the over-expression of microRNA-1 (miR-1) can target the 3′ UTRs of mitochondrial inner membrane organizing system 1 (MINOS1) and glycerol-3-phosphate dehydrogenase 2 (GPD2) under the participation of LRPPRC, which results in mitochondrial damage (Zhang et al., 2019). However, similar effects upon m^6^A RNA methylation need to be proved.

m^6^A RNA methylation is a complex regulative system that acts through methylation and demethylation on different targets. Its effect is also reflected in “promoter” or “suppressor” in tumorigenesis and development. With the improvement of RNA methylation detection technology, an increasing number of regulators are found, which reveals a more complicated regulation system. Further studies are needed to be done to verify these findings and their interactive mechanisms. Besides, the way to control specificity and to strengthen monitoring of RNA methylation regulators in the dynamic post-transcriptional balance is also a great challenge. But we believe, as a promising research area in epigenetics, RNA methylation would bring a new dawn of hope for combating breast cancer.

## Data Availability Statement

Publicly available datasets were analyzed in this study. This data can be found here: http://www.cancergenome.nih.gov/ and https://www.ncbi.nlm.nih.gov/geo/query/acc.cgi?acc=GSE70905.

## Author Contributions

GJ supervised and conceptualized the study. BZ searched the articles and designed the study. BZ and YG made the figures and wrote this manuscript. All authors read and revised the final manuscript.

## Conflict of Interest

The authors declare that the research was conducted in the absence of any commercial or financial relationships that could be construed as a potential conflict of interest.

## Footnotes

1 https://www.oncomine.org/

2 http://www.cancergenome.nih.gov/

3 https://www.ncbi.nlm.nih.gov/geo/

4 http://kmplot.com/analysis/

5 http://bcgenex.centregauducheau.fr/

6 http://string-db.org

7 https://www.gtexportal.org/

8 http://www.r-project.org/
