# Supplementary material for: Expression and Prognostic Characteristics of m6 A RNA Methylation Regulators in Breast Cancer
Source: Front Genet. 2020 Dec 10;11:604597. doi: 10.3389/fgene.2020.604597 (PMC7758326; doi:10.3389/fgene.2020.604597)
Supplement: Supplementary file 1 [file Presentation_1.pdf]

Supplementary Material

1 Supplementary Figures and Tables

1.1 Supplementary Figures

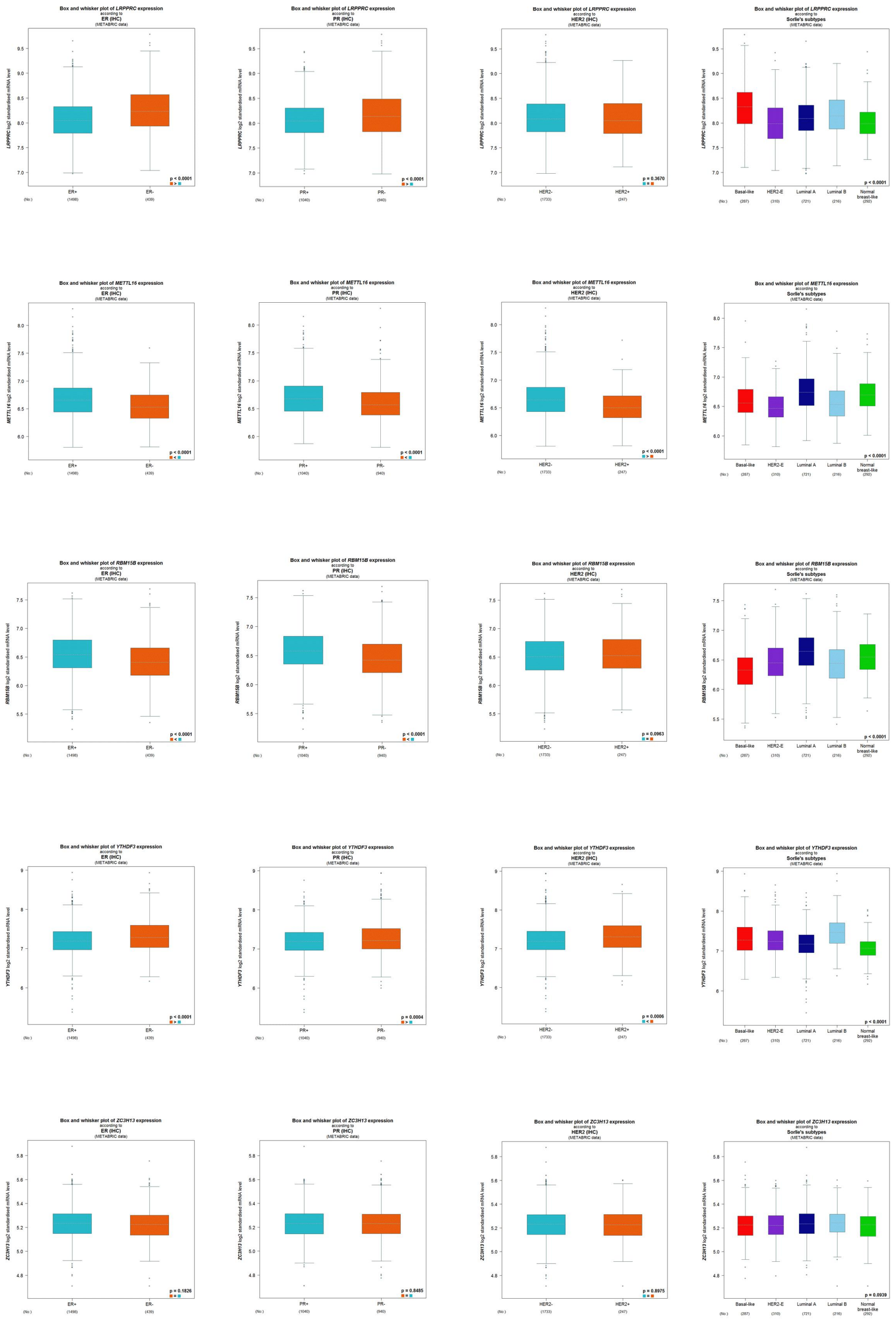

Supplementary Figure 1. METABRIC data from bc-GenExMiner v4.5 showing the comparison outcomes with ER, PR, HER-2 and PAM50 subtypes on the expression level of YTHDF3, RBM15B, ZC3H13, LRPPRC and METTL16.

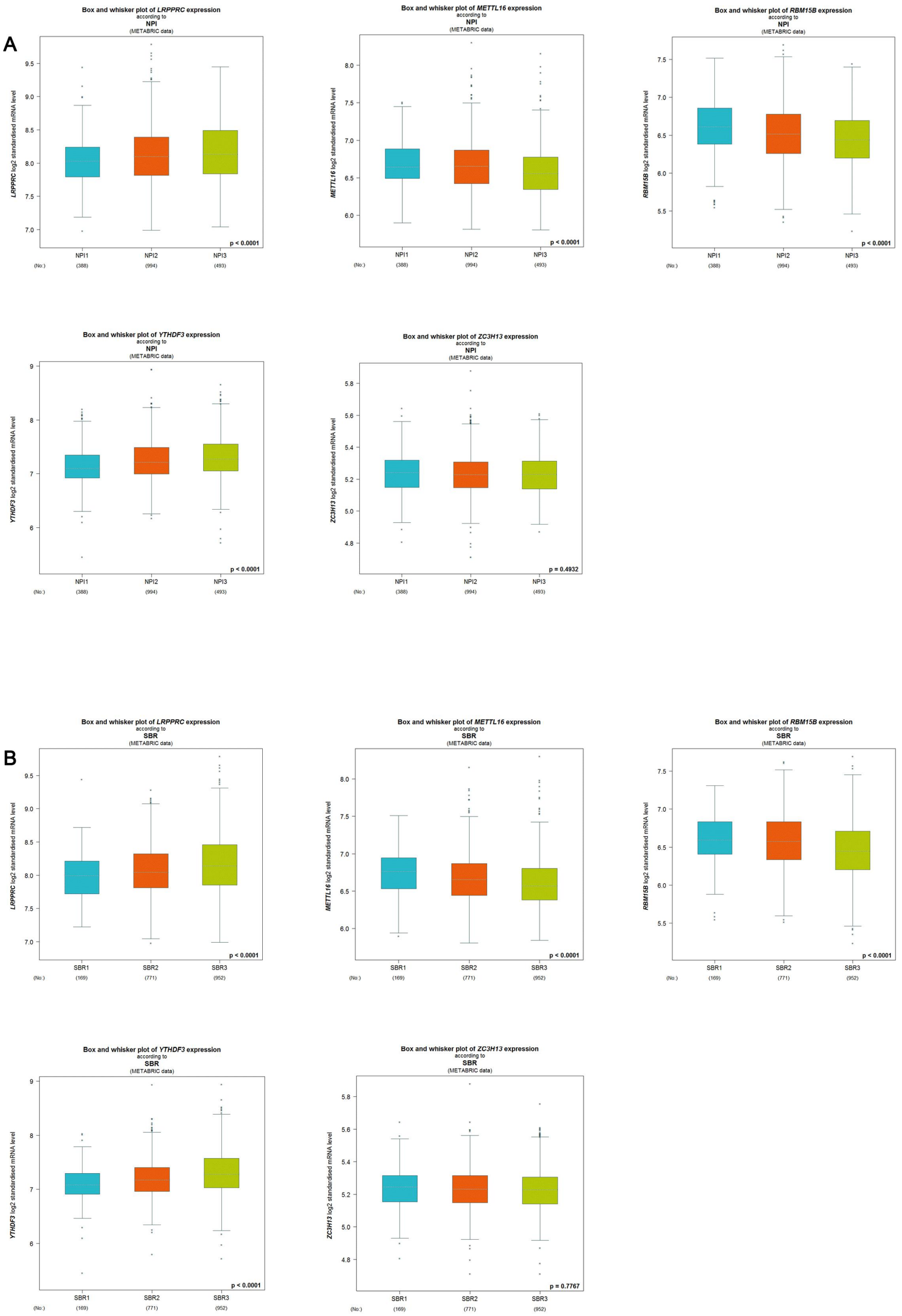

Supplementary Figure 2. The correlation between the expression level of LRPPRC, METTL16, RBM15B, YTHDF3 and ZC3H13 and the Nottingham Prognostic Index (NPI) **(A)** and the Scarff-Bloom-Richardson (SBR) **(B)** grading.
